# Association between Polymorphisms in Glutathione Peroxidase and Selenoprotein P Genes, Glutathione Peroxidase Activity, HRT Use and Breast Cancer Risk

**DOI:** 10.1371/journal.pone.0073316

**Published:** 2013-09-10

**Authors:** Catherine Méplan, Lars Ove Dragsted, Gitte Ravn-Haren, Anne Tjønneland, Ulla Vogel, John Hesketh

**Affiliations:** 1 Institute for Cell and Molecular Biosciences, Newcastle University, Newcastle-upon-Tyne, United Kingdom; 2 Human Nutrition Research Centre, Newcastle University, Newcastle-upon-Tyne, United Kingdom; 3 Department of Human Nutrition, University of Copenhagen, Frederiksberg, Denmark; 4 National Food Institute, Technical University of Denmark, Soborg, Denmark; 5 Danish Cancer Society Research Centre, Copenhagen, Denmark; 6 National Research Centre for the Working Environment, Copenhagen, Denmark; University of North Carolina School of Medicine, United States of America

## Abstract

Breast cancer (BC) is one of the most common cancers in women. Evidence suggests that genetic variation in antioxidant enzymes could influence BC risk, but to date the relationship between selenoproteins and BC risk remains unclear. In this report, a study population including 975 Danish cases and 975 controls matched for age and hormone replacement therapy (HRT) use was genotyped for five functional single nucleotide polymorphisms (SNPs) in *SEPP1, GPX1, GPX4* and the antioxidant enzyme *SOD2* genes. The influence of genetic polymorphisms on breast cancer risk was assessed using conditional logistic regression. Additionally pre-diagnosis erythrocyte GPx (eGPx) activity was measured in a sub-group of the population. A 60% reduction in risk of developing overall BC and ductal BC was observed in women who were homozygous Thr carriers for *SEPP1* rs3877899. Additionally, Leu carriers for *GPX1* Pro198Leu polymorphism (rs1050450) were at ∼2 fold increased risk of developing a non-ductal BC. Pre-diagnosis eGPx activity was found to depend on genotype for rs713041 (*GPX4*), rs3877899 (*SEPP1*), and rs1050450 (*GPX1*) and on HRT use. Moreover, depending on genotype and HRT use, eGPx activity was significantly lower in women who developed BC later in life compared with controls. Furthermore, GPx1 protein levels increased in human breast adenocarcinoma MCF7 cells exposed to β-estradiol and sodium selenite.In conclusion, our data provide evidence that SNPs in *SEPP1* and *GPX1* modulate risk of BC and that eGPx activity is modified by SNPs in *SEPP1, GPX4* and *GPX1* and by estrogens. Our data thus suggest a role of selenoproteins in BC development.

## Introduction

Breast cancer (BC) is a major cause of death in the Western societies [1]. Inherited *BRCA1* and *BRCA2* mutations account for a significant proportion (5–10%) of all breast cancer cases among white women in the United States [2]. Despite identification of additional risk factors such as endocrine factors, alcohol and body mass index [3], [4], much of the aetiology of BC remains unclear. It has been suggested that generation of oxidative stress within breast tissue may play a role in cancer initiation and development by causing DNA damage and mutations [5], [6]. On the basis of this hypothesis several studies have investigated whether genetic polymorphisms in antioxidant enzymes influence either susceptibility to BC or prognosis after diagnosis [7], [8], [9], [10].

One focus of such studies is the genes encoding the selenocysteine-containing selenoproteins. Selenoproteins, which carry out the major biological activity of micronutrient selenium (Se), are potentially important with respect to BC development because they protect cells from damaging free radicals [11] and because Se status, as assessed by serum Se concentration, has been shown to decrease in BC patients compared with healthy controls [12], [13]. Glutathione peroxidases 1 (GPx1) and 4 (GPx4) are well characterised antioxidant selenoenzymes that detoxify peroxide radicals and lipid hydroperoxides, respectively [14]. Selenoprotein P (SePP) exhibits both Se transport and antioxidant functions [15]. These three genes contain common genetic functional polymorphisms. Rs1050450 in the *GPX1* gene causes an amino acid change from Pro to Leu at codon 198 [16], with the Leu variants being less active than its Pro counterpart [17]. Rs713041 in *GPX4* affects protein binding to the 3′untranslated region (3′UTR) of the mRNA close to an RNA structure (selenocysteine insertion sequence: SECIS) required for selenoprotein synthesis, although the nature of the proteins affected has not been defined [18], [19], [20]. The human SePP gene (*SEPP1*) contains several functional polymorphisms, including rs3877899 (Ala234Thr) and rs7579 (a G/A base change in the 3′UTR of *SEPP1* mRNA) which affect plasma and lymphocyte selenoprotein activity *in vivo* and the relative proportion of plasma SePP isoforms [21], [22]; in addition both SNPs have also been reported to be associated with colorectal and prostate cancer risk [23], [24], [25]. A small number of functional variants have been reported in other selenoprotein genes [18] and in non-selenoprotein genes such as rs4880 in the *SOD2* gene encoding antioxidant manganese superoxide dismutase (MnSOD) [23], [24], [26].

To date, the study of selenoprotein genetic variants in relation to BC risk has been limited. A higher frequency of the Leu variant of rs1050450 (*GPX1*) was found in breast tumour DNA compared to normal tissue, probably due to loss of heterozygosity in the tumour cells [17]. Two subsequent genetic association studies failed to identify an association of rs1050450 alone with BC risk [9], [27], but risk was increased in individuals carrying both the Leu variant in *GPX1* and the Ala variant for rs4880 (*SOD2*) [28]. In addition, carriage of the Leu variant for rs1050450 in combination with alcohol intake was associated with higher BC risk [10]. Thus, it is still unclear whether rs1050450 (*GPX1*) is a risk factor for BC [29]. Additionally, an association of rs713041 (*GPX4*) genotype and disease prognosis has been reported [26]. However, no genetic association study of genotype for either rs713041 (*GPX4*) or for any SNPs in *SEPP1* and BC risk has been carried out so far, although lymphocyte GPx4 mRNA expression was found to be lower in breast cancer patients than controls [30].

On the basis of the key role of Selenoprotein P in Se transport and the functions of GPx1, GPx4 and MnSOD in antioxidant defence mechanisms, we hypothesize that functional genetic variants in the corresponding genes influence the susceptibility to BC. To address this we focussed on known functional SNPs in these genes and genotyped BC patients and controls from the Danish prospective 'Diet, Cancer and Health Study', expanding on an earlier report [10] by increasing both the number of participants and the number of genetic variants genotyped, and by including tumour histology and grade in the analysis.

## Materials and Methods

### Study design and participants

The subjects for the present study were selected from the ongoing Danish “Diet, Cancer and Health” cohort study [31]. Between December 1993 and May 1997, 79,729 women aged 50 to 64 years, born in Denmark, living in the Copenhagen or Aarhus areas and having no previous cancers at this time were invited to participate in the study. A total of 29,875 women (37%) accepted the invitation. Cohort members were followed up for diagnosis of BC from date of entry until either the date of diagnosis of any cancer (except for non-melanoma skin cancer) using record linkage to the Danish Cancer Registry up to 2003 or afterwards with record linkage to the Danish Pathology Databank, date of death, date of emigration, or April 27^th^, 2006, whichever came first. The study group comprised a total of 975 women diagnosed with BC during a follow-up period until April 27**^th^**, 2006 [32], [33] and included 377 previously described postmenopausal women with breast cancer and matched controls identified at follow-up until 2003 [10], [33], [34], [35]. For each case with diagnosed BC, one matched control was selected [32]. The control was cancer-free at the exact age at diagnosis of the case and was further matched on age at inclusion into the cohort (half-year intervals) and on use of hormone replacement therapy (HRT) (current/former/never). For the cases, tumour classification according to grade (ductal grade 1, 2 or 3) or histology (ductal or non-ductal) was carried out following criteria recommended by Danish Breast Cancer Cooperative Group (see [36]). Total Se intake was estimated from food frequency questionnaire data on dietary intake and use of supplements [10]. Alcohol intake was calculated from the food frequency questionnaire data as described previously [32].

The Diet, Cancer and Health study and the present sub-study were approved by the regional Ethical Committees on Human Studies in Copenhagen and Aarhus (jr.nr.(KF)11-037/01) and (jr.nr.(KF)01-045/93), and by the Danish Data Protection Agency. All participants gave written consent, and the procedure was approved by the regional Ethical Committees on Human Studies in Copenhagen and Aarhus.

### Blood sampling and storage

30 ml blood were collected from non-fasting participants at the time of recruitment. Plasma, serum, lymphocytes, and erythrocytes were isolated and frozen at −20°C within 2 hours. At the end of the day of collection, all samples were stored in liquid nitrogen. Erythrocyte GPX activity was determined previously [10].

### Genotyping

DNA was isolated from frozen lymphocytes as previously described [37]. Generally, 100 μg DNA were obtained from 10^7^ lymphocytes. Twenty ng of DNA was genotyped in 5 ul containing 1x Mastermix (Applied Biosystems), 100 nM probes, and 900 nM primers and analysed by allelic discrimination on an ABI7900HT (Applied Biosystems). Controls with known genotypes were included in each run, and repeated genotyping of 10% yielded 100% identical genotypes. Genotyping of 377 pairs for GPX1 Pro198Leu (rs1050450) has been published previously [10] and extended to the whole cohort. Primers and probes for the other SNPs were as follows:*SOD2* (rs4880) Val16Ala PCR forward; 5′-GGC TGT GCT TTC TCG TCT TCA-3′, PCR reverse: 5′-CAT GAT CTG CGC GTT GATG-3′, probes: T-allele: 5′-^FAM^-CTC CGG TTT TGGG-^MGB^-3′ and C-allele: 5′-^VIC^-CTC CGG CTT TGGG-^MGB^-3′; *GPX1* (rs1050450) was as previously published; PCR forward, 5′-TGT GCC CCT ACG CAG GTA CA-3′; PCR reverse, 5′-CCC CCG AGA CAG CAG CA-3′; C allele probe, 5′-^VIC^CTG TCT CAA GGG CCC AGC TGTG C^TAMRA^; and T allele probe, 5′-^FAM^CTG TCT CAA GGG CTC AGC TGT GCCT^TAMRA^-3′ [38]; *GPX4* (rs713041) PCR forward: 5′-CCC ACT ATT TCT AGC TCC ACA AGTG-3′ and PCR reverse 5′-GTC ATG AGT GCC GGT GGAA-3′, probes were T-allele: 5′-^FAM^-ACG CCC TTG GAGC -^MGB^-3′ and C-allele: 5′-^VIC^-ACG CCC TCG GAGC-^MGB^-3′; *SEPP1*; rs3877899 was determined using a premade Taqman assay (Assay 2841533_10 Applied Biosystems) and rs7579 primers were: PCR forward: 5-‘CAA AAA AGT GAG AAT GAC CTT CAA ACT-3’ and PCR reverse: 5′-ATG CTG GAA ATG AAA TTG TGT CTA GA-3′, probes G-allele: 5′-^VIC^-AAA ATA GGA CAT ACT CCC C-^MGB^-3′ and A-allele: 5′-^FAM^-AAA TAG AAC ATA CTC CCC AAT T-^MGB^-3′ (MGB, minor groove binder; FAM, carboxyfluorescein; TAMRA, carboxytetramethylrhodamine).

### Cell culture and preparation of protein extracts

MCF7 human breast adenocarcinoma cells were maintained at 37°C and 5% CO_2_ in Dulbecco's Modified Eagles Medium (DMEM; Gibco, Invitrogen, DMEM-GlutaMAX), supplemented with 10% foetal bovine Serum (Sigma), 1% Non Essential Amino Acids (Gibco, Invitrogen) and penicillin/streptomycin (Invitrogen; 100U/500ml media). Cells were treated with Dimethyl sulphoxide (DMSO, Sigma) (negative control), DMSO + sodium selenite (7 ng/ml; 40 nM, Sigma) (corresponding to Se-adequate conditions and referred as Se-supplemented in the text by contrast with the DMEM medium which is poor in Se), β-estradiol (10nM prepared in DMSO), or a combination of 40 nM sodium selenite and 10 nM β-estradiol. After 48h treatment, cells were washed twice in ice-cold PBS, lysed and scraped into130 μl of lysis buffer (25mM HEPES buffer pH7.6, 3 mM MgCl_2_, 40 mM KCL, 5% glycerol, 0.5% NP-40, 2 mM DTT) in the presence of a cocktail of protease inhibitors (Roche). Cells were sonicated on ice 3 times for 20 sec (Soniprep 150, Sanyo), centrifuged for 5 mins at 13,000 rpm at 4°C and the supernatant collected. Protein concentration was determined by Bradford assay (Sigma).

### Sodium dodecyl sulphate (SDS)-polyacrylamide gel electrophoresis and Western-blotting

Proteins (40 μg) were separated by electrophoresis on a 10% SDS-polyacrylamide gel and electro-transferred onto a PVDF membrane (Roche). Membranes were blocked overnight in PBS containing 0.05% Tween 20 and 5% dried skimmed milk at 4°C and then incubated at room temperature for 1 h with polyclonal anti-polyclonal GPx1 antibody at a 1:500 dilution in PBS containing 0.05% Tween 20 and 5% dried skimmed milk (Abcam) or with anti-β-actin (1:5,000 dilution, Sigma). Membranes were then washed 5 times in PBS-0.05% Tween 20, and incubated for 1 h with HRP-conjugated secondary antibodies, either anti-rabbit antibody (1:5000 dilution, Sigma) for anti-GPx1 or anti-mouse (1:5000 dilution, Sigma) for anti-β-actin. After a further 5 washes of the membranes, immuno-detection was carried out using a chemiluminescence kit (GE Healthcare) on Amersham HyperfilmTM ECL (GE Healthcare). Band intensity was quantified using UVIband software (Uvitec, UK).

### Statistical analysis

To estimate the association between individual SNPs and breast cancer, we calculated odds ratios (OR) and 95% confidence intervals (95% CI) using conditional logistic regression analysis and STATA 11.0 statistical software. Estimates were adjusted for age, body mass index (BMI), smoking, fruit and vegetable intake, alcohol and tobacco consumption, HRT use, number and year of childbirths and Se intake estimated from a food frequency questionnaire. Genotypes were evaluated using indicator variables with the common homozygote as reference. Two models were tested: a recessive model, in which each genotype was compared with the homozygote for the frequent allele to assess the effect of each genotype on the risk of breast cancer and a dominant model in which heterozygotes and homozygotes for the rare allele were pooled together and compared with the homozygote for the frequent allele to assess the effect of the presence of at least one rare allele on breast cancer risk. Conditional logistic regression of two-loci interaction was performed for selected SNPs when either (i) the SNPs showed a main effect or (ii) multivariate conditional logistic regression suggested an interaction and when in addition, the likelihood ratio test showed significant interactions (*P*≤0.05). ORs and 95% CI are presented with reference to the double homozygous genotype for the most frequent allele.

## Results

### Genotype, tumour classification and disease risk

The overall study population included 975 cases and 975 controls matched for age and hormone replacement therapy (HRT). Baseline characteristics of women diagnosed with breast cancer and their matched controls are presented in Table 1 (means and 95% confidence interval) and findings regarding all the included risk factors have been reported previously [32], [33], [34].

**Table 1:** Baseline characteristics of the study population.

Variable	Cases (*N* = 975)	Controls (*N* = 975)
**Mean age [95%CI]**	57.2 [56.9–57.4]	57.2 [56.9–57.4]
**Present smoking (%)**	23.1	22.6
**Mean Body mass index [95%CI]**	25.4 [25.1–25.7]	25.7 [25.2–25.7]
**Ductal breast cancer**	659 (67.6%)	
*Grade 1*	267 (27.4%)	
*Grade 2*	265 (27.2%)	
*Grade 3*	127 (13%)	
**Non ductal breast cancer**	190 (19.4%)	
**Tumour Classification Unknown**	126 (13%)	
**HRT use**		
*Never*	332 (34%)	332 (34%)
*Current*	519 (53.2%)	519 (53.2%)
*Former*	124 (12.8%)	124 (12.8%)
**Duration of HRT use in years (among current users)**	6.6[6.1–7]	6.9[6.4–7.4]
**School education**		
*Low (%)*	302 (31%)	341 (35%)
*Medium (%)*	476 (48.9%)	461 (47.4%)
*High (%)*	196 (20.1%)	171 (17.6%)
**Parous (%)**	84.8	87.8
**Mean Number of births [95%CI]**	1.8 [1.7–1.9]	1.9 [1.9–2]
**Mean Age at first birth, years [95%CI]**	23.9 [23.6–24.2]	23.5 [23.16–23.7]
**Mean alcohol intake in g/day [95%CI]**	15.3 [13.7–16.9]	14.1 [12.8–15.5]
**Mean selenium intake in μg/day [95%CI]**	65.1 [63.1–67.2]	67.8 [65.3–70.3]

Genotype frequencies for rs4880 (*SOD2*), rs7579 and rs3877899 (*SEPP1*), rs713041 (*GPX4*) and rs1050450 (*GPX1*) are shown in Table 2. With the exception of rs713041, the genotype distributions of the studied polymorphisms were in Hardy-Weinberg equilibrium (HWE) among the controls. The failure of rs713041 to conform to HWE may reflects the fact that controls were matched for secondary criteria including HRT and thus do not represent the general population [39]. To address the hypothesis that known functional variants in selenoprotein genes affect BC risk and tumour grade or histology the association of the individual SNPs with BC risk was assessed by conditional logistic regression using recessive and dominant models and adjusted as described in the method section. Genotype for *SEPP1* Ala234Thr (rs3877899) was significantly associated with risk of breast cancer with the homozygous AA genotype (Thr/Thr) having a lower risk of breast cancer (OR 0.39, 95%CI 0.2–0.75, p = 0.005, Table 2).

**Table 2:** Effect of selenoprotein SNPs on breast cancer risk.

*GENE* (polymorphism)	Genotype	Cases/ Controls	OR (95% CI)	p-value
*SOD2* (rs4880)	Total	939/958		
	CC	226/227	1 [-]	
	CT	485/494	1.09 [0.83–1.45]	0.53
	TT	228/237	0.96 [0.69–1.34]	0.813
	CT+TT	713/731	1.05 [0.8–1.38]	0.701
*GPX1 *(rs1050450)	Total	933/959		
	CC	465/503	1 [-]	
	CT	396/370	1.03 [0.81–1.32]	0.789
	TT	72/86	0.83 [0.54–1.28]	0.393
	CT+TT	468/456	0.99 [0.79–1.25]	0.96
*GPX4* (rs713041)	Total	939/960		
	CC	319/335	1 [-]	
	CT	438/430	1.16 [0.9–1.49]	0.241
	TT	182/195	0.92 [0.66–1.28]	0.63
	CT+TT	620/625	1.09 [0.86–1.38]	0.459
*SEPP1* (rs3877899)	Total	937/959		
	GG	586/594	1 [-]	
	GA	321/317	1 [0.78–1.28]	0.984
	AA	30/48	***0.39 [0.2–0.75]***	***0.005***
	GA+AA	351/365	0.91 [0.72–1.15]	0.436
*SEPP1* (rs7579)	Total	937/957		
	GG	455/436	1 [-]	
	GA	396/420	0.86 [0.68–1.09]	0.206
	AA	86/101	0.75 [0.5–1.13]	0.163
	GA+AA	482/521	0.84 [0.67–1.05]	0.125

Adjusted values were analysed by conditional logistic regression. Odd ratios (95% confidence interval) and p-values are presented together with the number of cases and controls for each genotype. Significant results are presented in bold and italic.

BC cases were classified as having either ductal tumours (659) with 267 being grade 1, 265 grade 2 and 127 grade 3 or non-ductal tumours (190 cases); 126 cases had no tumour classification at the time of follow up. Stratifying the genotyping data according to these diagnostic criteria allowed analysis of the association of genotype with risk of particular tumour grade or histology (Table 3). Rs3877899 (*SEPP1*) was associated with risk of ductal tumours with homozygous AA having a lower disease risk (OR 0.48, 95% CI 0.26–0.88, p = 0.017). In contrast, rs3877899 was not associated with risk of a non-ductal tumour; however, women homozygous TT for rs1050450 (*GPX1*, Leu variant) had an increased risk of having a non-ductal tumour (1.88 [1.08–3.28], p = 0.02*7*) but it should be noted that there were few individuals with this genotype. In addition, Leu carriers (rs1050450, *GPX1*) had an increased risk of having a grade 3 ductal tumour compared with grade 1 and 2 (OR 2.64, 95%CI 1.13–6.16 p = 0.025). None of the other SNPs were associated with BC or BC subtypes.

**Table 3:** Effect of selenoprotein SNPs on breast cancer risk after stratification of data according to tumourgrade and histology.

		Ductal vs control		Non-ductal vs control	
*GENE (polymorphism)*	Genotype	OR (95% CI)	p-value	OR (95% CI)	p-value
*SOD2 (rs4880)*	CC	1[-]		1[-]	
	CT	0.98 [0.74–1.29]	0.88	1.13 [0.72–1.77]	0.604
	TT	0.99 [0.72–1.36]	0.937	1.18 [0.7–1.99]	0.525
*GPX1 (rs1050450)*	CC	1[-]		1[-]	
	CT	1.16 [0.92–1.47]	0.206	1.13 [0.77–1.66]	0.545
	TT	0.73 [0.47–1.14]	0.166	***1.88 [1.08–3.28]***	***0.027***
*GPX4 (rs713041)*	CC	1[-]		1[-]	
	CT	1.16 [0.9–1.5]	0.237	1.02 [0.68–1.52]	0.935
	TT	0.91 [0.66–1.25]	0.552	1.03 [0.63–1.67]	0.911
*SEPP1 (rs3877899)*	GG	1[-]		1[-]	
	GA	0.98 [0.77–1.24]	0.849	0.92 [0.62–1.35]	0.656
	AA	***0.48 [0.26–0.88]***	***0.017***	0.89 [0.4–1.99]	0.785
*SEPP1 (rs7579)*	GG	1[-]		1[-]	
	GA	0.91 [0.72–1.15]	0.42	1.17 [0.81–1.69]	0.404
	AA	0.86 [0.58–1.28]	0.454	0.65 [0.32–1.33]	0.235

Adjusted values were analysed by conditional logistic regression. Odd ratios (95% confidence interval) and p-values are presented for each genotype. Significant results are presented in bold and italic.

Selenoprotein P supplies Se to non-hepatic tissues [40] to support synthesis of other selenoproteins and therefore we tested the hypothesis that *SEPP1* variants interacted with SNPs in other selenoprotein genes to modify BC risk by first carrying out a multivariate logistic regression including all 5 SNPs. Using a dominant model stratification of the genotyping data according to tumour grade or histology revealed an interaction between rs3877899 (*SEPP1*) and rs1050450 (*GPX1*), with women who were heterozygous for both rs1050450 (CT) and rs3877899 (GA) being at increased risk of developing non-ductal breast cancer (OR = 2.94, 95%CI 1.35–6.37, p = 0.006, Table 4). Similar results were obtained using a dominant model, with women carrying both rs1050450 (CT+ TT) and rs3877899 (GA +AA) having an increased risk of developing non-ductal breast cancer (OR = 2.59, 95%CI = 1.28–5.22, p = 0.008, Table 4). In addition, we observed a genetic interaction between rs1050450 (*GPX1*) and rs7579 (*SEPP1*), with carriers of both rs1050450 (CT+TT) and rs7579 (GA+AA) genotype having an increased risk of a grade 3 tumour compared with grades 1 and 2 (OR = 2.63, 95%CI = 1.63–6.71, p = 0.042, Table 4), indicating that the association of rs1050450 genotype with the risk of grade 3 tumour was modified by genotype for rs7579 in *SEPP1*. Although carriage of only one copy of the Leu allele (*GPX1*) showed no statistical effect on disease risk, the influence of heterozygosity for rs1050450 was modified by both *SEPP1* SNPs, with heterozygosity for both rs1050450 and rs3877899 increasing the risk (OR = 3.36, 95%CI 1.43–7.94, p = 0.006, Table 4) but heterozygosity for both rs1050450 and rs7579 reducing the risk (OR = 0.34, CI 0.15–0.77, p = 0.01, Table 4). It was not possible to analyse effects of interactions of these other variants with being homozygous for the Leu allele of rs1050450 because of the small numbers of individuals with those combined genotypes. However, taken together the observation that being homozygous for the rs1050450 Leu allele significantly increases risk of a non-ductal tumour combined with the significant effects of heterozygosity for rs1050450 and rs3877899 on risk of the same diagnosis indicates that carriage of at least a minor Leu allele is associated with increased risk of such a tumour.

**Table 4:** Significant SNP-SNP interactions in relation to breast cancer risk and tumour grades.

SNP-SNP interaction	Genotype combination	Compared groups	OR [95% CI]	p-value
rs1050450 (GPX1) *rs7579 (SEPP1)	(CT+TT) * (GA+AA)	ductal grade 3 (43/39)/ductal grade 1 &2(127/154)	2.63 [1.63–6.71]	0.042
rs1050450 (GPX1) * rs38773899 (SEPP1)	(CT) * (GA)	non-ductal (31/63)/ductal(91/193)	2.94 [1.35–6.37]	0.006
rs1050450 (GPX1) * rs38773899 (SEPP1)	(CT+ TT) * (GA +AA)	non-ductal(42/63)/ductal(110/193)	2.59 [1.28–5.22]	0.008
rs1050450 (GPX1) * rs38773899 (SEPP1)	(CT)*(GA)	non-ductal(31/63)/control (166/306)	3.36 [1.43–7.94]	0.006
rs1050450 (GPX1) * rs38773899 (SEPP1)	(CT+TT)*(GA+AA)	non-ductal (42/63)/control (121/306)	2.77[1.37–6.2]	0.006
rs1050450 (GPX1) * rs7579 (SEPP1)	(CT)*(GA)	non-ductal (23/35)/control(172/231)	0.34 [0.15–0.77]	0.01

Only significant interactions between two loci, as identified by logistic regression, are presented. Loci are identified by rs number and OR values with 95% CI are shown as well as p-values. Compared group are indicated with the ratio corresponding to the number of women with the combined genotypes as indicated in the adjacent left column/number of women double homozygous for the reference most frequent allele.

### Genotype, GPx activity and disease risk

**Figure 1:**
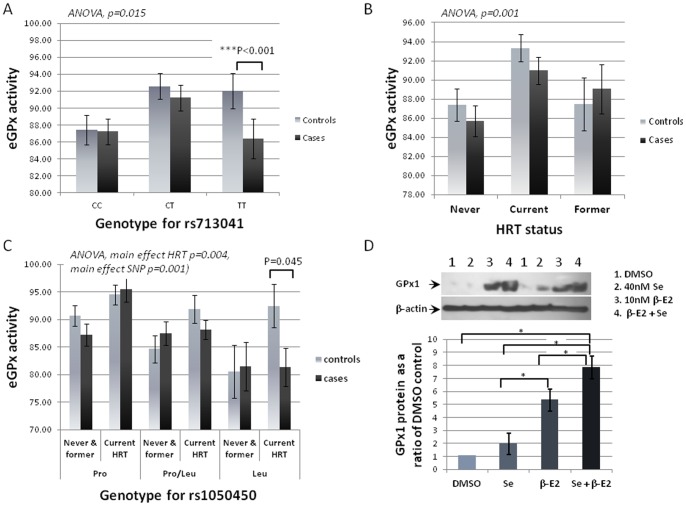
Regulation of GPx activity and protein levels. (A) shows the association of rs713041 genotype (*GPX4*) with pre-diagnosis erythrocyte GPx (eGPx) activity (*Anova, p = 0.015*). EGPx activity was lower in TT women who developed BC at a later stage in life. (B) eGPX activity was higher activity in women using HRT (*Anova, p = 0.001*). No differences were observed between cases and controls. (C) Combination of HRT use and rs1050450 in *GPX1* affects eGPX activity (*Anova, main effects of HRT p = 0.004, rs*1050450 *p = 0.001*). HRT use increases eGPX activity in all controls, but not in Leu carriers who developed BC at a later stage in life. (D) MCF7 cells were exposed to 10nM β-estradiol (β-E2) ± 40nM sodium selenite (Se). Western blotting of total cell protein revealed as significant increase in GPx1 protein level when cells were treated with β-E2 compared with Se, Se+ β-E2 compared with β-E2 alone or Se alone (Kruskal-Wallis test, effect of treatment p = 0.005; Mann Whitney test* = p<0.05). Values shown are means ± SEM (n = 3).

Erythrocyte GPx activity (eGPx), which largely reflects GPx1 expression, is influenced by rs1050450 (*GPX1*) genotype [41], [42]. Pre-diagnostic eGPX activity was available for 377 matched pairs in the study population and since selenoproteins essentially compete for available Se during synthesis, we tested the hypothesis that SNPs in other selenoprotein genes modulate eGPx activity. Univariate analysis revealed a main effect of rs713041 in *GPX4* on eGPx activity (p = 0.015), with CC carriers having lower activity than CT or TT carriers (Figure 1A). In addition, TT carriers for rs713041 who later develop BC exhibited a significantly lower eGPx compared with the ones who did not develop the disease (p<0.001). This effect did not reflect a difference between controls and cases in eGPx activity (data not shown, p = 0.257). These effects of the *GPX4* variant on eGPx activity are compatible with previous work showing that rs713041 affects blood cell GPx1 protein levels, probably by affecting the ability of the *GPX4* mRNA to compete in the selenoprotein synthesis hierarchy [19].

In addition, current HRT users at the time of eGPx measurement exhibited higher activity than women who had either never or previously used HRT (ANOVA, main effect HRT p = 0.001, Figure 1B) independently of the BC status. However when genotype for rs1050450 was taken into account, we observed a main effect of HRT (ANOVA, p = 0.004) and of rs1050450 (ANOVA, p = 0.001) in the prediction of eGPx activity level. Current HRT users (both cases and controls) who were Pro or Pro/Leu carriers showed higher eGPx activity compared with never or former HRT users (Figure 1C). On the contrary, eGPx activity was increased in homozygous Leu carrier controls who were current HRT users but not among Leu carriers who at a later stage in their life developed breast cancer (p = 0.045; Figure 1C).

The observed effects of HRT on eGPx activity suggested that GPx1 expression is regulated by estrogen. To test this hypothesis, MCF7 cells were treated with 10 nM β-estradiol for 48 h in the presence or absence of sodium selenite (40 nM) and GPx1 protein level (relative to β-actin control) assessed by western-blot analysis (Figure 1D). GPx1 protein levels were very low in cells grown in standard medium but supplementation with sodium selenite resulted in a 1.98 (±0.82) fold increase of GPx1 protein after 48h. β-estradiol treatment resulted in a larger increase of GPx1 protein level (5.33 (±0.83) fold) and this effect was additive in the presence of the combination β-estradiol and sodium selenite (7.84 (±0.85) fold).

## Discussion

The aetiology of breast cancer is only partially understood but oxidative stress-induced DNA damage has been suggested to be critical for the initiation and progression of the disease [5], [43]. Several selenoproteins are known to have antioxidant functions [11] and the present work provides evidence that genetic variants in *SEPP1* and *GPX1* affect BC risk.

A dramatic 60% decrease in risk of developing total or ductal BC was observed in homozygous Thr carriers for rs3877899 (*SEPP1*) compared with Ala carriers. Since there was no observed effect of this variant on risk of a non-ductal tumour it is likely that the decreased risk of developing total BC due to this SNP is largely due to an effect on developing a ductal tumour. To our knowledge, this is the first evidence that this SNP influences BC risk. SePP has a major role in Se transport [40] and previous data have indicated that both rs3877899 and rs7579 in *SEPP1* modulate selenoprotein concentrations and activities in the plasma, erythrocytes and lymphocytes and plasma SePP isoform pattern [21], [22], suggesting that these polymorphisms can affect Se delivery to tissues. The effects of genotype for rs3877899 (*SEPP1*) on BC risk suggest that altered Se supply to the breast tissue could contribute to BC development, potentially by affecting local expression of other selenoproteins.

**Figure 2:**
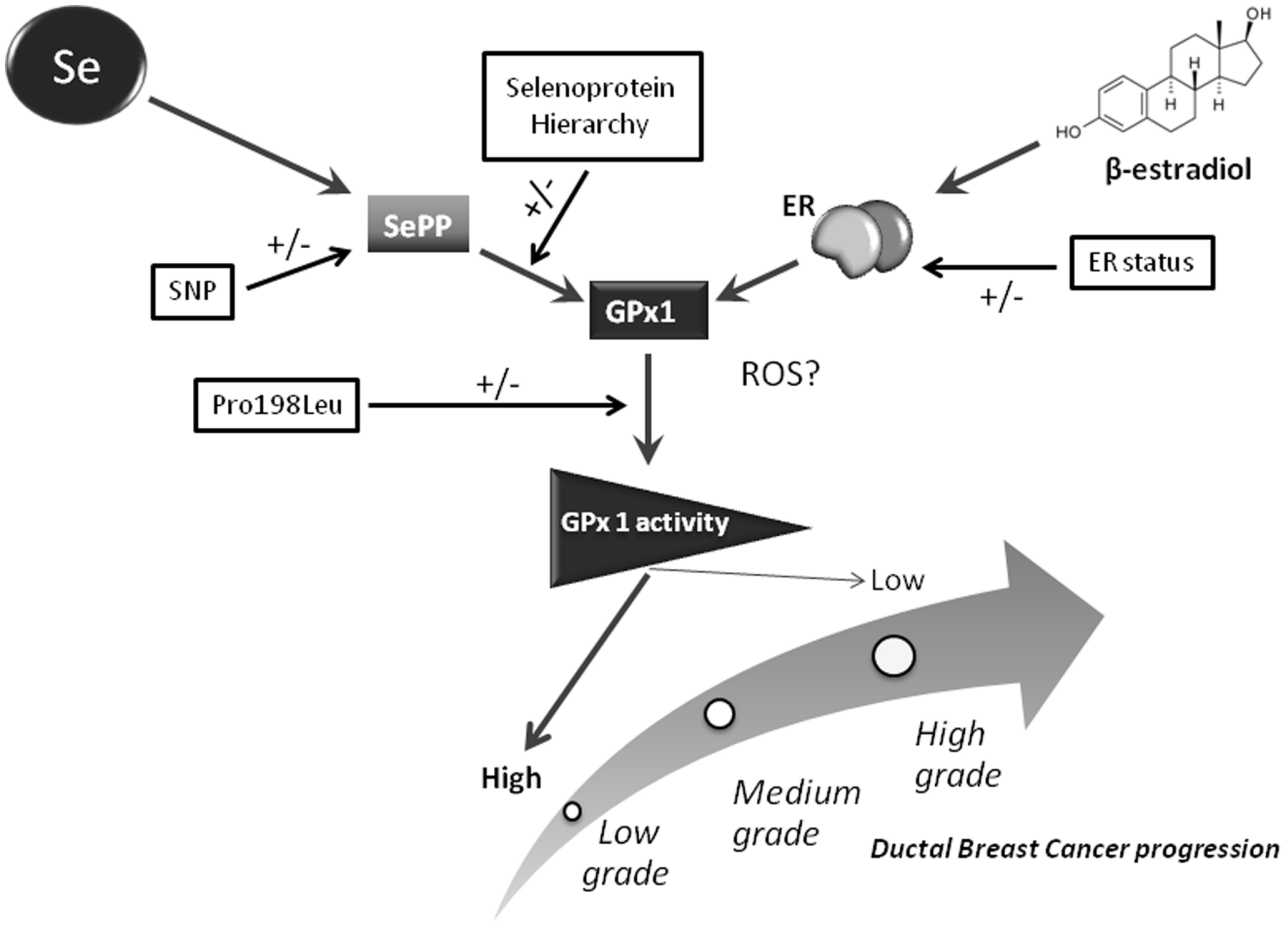
Model of the regulation of GPx1 in breast cells by selenium and estrogens. Our hypothesis is that GPx1 concentration in breast cells is controlled by (i) factors that affect Se bioavailability (Se supply and polymorphisms in *SEPP1*) or the selenoprotein hierarchy (e.g. rs713041 in *GPX4*), (ii) estrogen levels and estrogen receptor status of breast tumour cells, (iii) the Pro198Leu polymorphism (rs1050450) in *GPX1*. Combinations of these factors contributing to low GPx1 activity/levels will result in lower capacity to respond to reactive oxygen species (ROS), favouring accumulation of oxidative damage and promoting tumour progression. In contrast maintaining high GPx1 activity has the potential to delay tumour progression.

Leu carriers for rs1050450 (Pro198Leu) in *GPX1* were shown to have a 1.9-fold increased risk of developing non-ductal breast cancer. In addition, carriers of this variant were also 2.6 fold more likely to have a grade 3 ductal tumour compared to a grade 1 or 2 tumour. Since enzymatic assays have previously shown that the Leu protein variant is less active than the Pro counterpart [16], [17] we propose that high GPx1 activity is required to counterbalance the levels of ROS and related damage occurring during initiation or progression of the disease (Figure 2).

Although a meta-analysis [44] indicated an association between BC risk and genotype for Pro198Leu (*GPX1*) in premenopausal women, a genome-wide association study (GWAs) approach failed to find any statistically significant associations for either rs1050450 (*GPX1*) or rs4880 (*SOD2*) [44], [45], [46]. In addition, in the GWAs rs3877899 (*SEPP1*) was not identified as being associated with BC risk, nor was it apparently linked to SNPs showing an association [46]. It is not clear why the present analysis revealed effects that were not observed from this GWAs carried out on a US population [44], [45], [46] but since generally Se intake is higher in US than in Europe [47] it is possible that effects of genotype for rs1050450 (*GPX1*) were revealed in the Danish cohort because of a lower, sub-optimal, Se intake. An estimate of Se intake was calculated on the basis of food questionnaires [48] for the present cohort (67.8 μg/day [65.3–70.3] in controls and 65.1 μg/day [63.1–67.2] in cases, Table 1) and indeed this suggests that their Se intake is lower than that found in the US (>100 µg/day). Thus, it is possible that the observed effects of the variants are particularly relevant to populations where Se intake in low. Interestingly, a GWAs carried out in UK identified a locus located close to the *SEPP1* gene to be associated with BC risk [49] but it is not known if it is linked to either rs3877899 or rs7579. In addition, the present study population was well characterised in terms of disease stage and HRT, and this may reduce confounding of genetic effects by such factors. Finally, alcohol intake is generally high amongst Danish women and this may modify the association of BC with SNPs in antioxidant enzymes, as observed previously [7], [8], [9], [10]. The mean alcohol intake in the present study (estimated from food frequency questionnaires) was 14.1 [12.8–15.5] g/day for controls and 15.3 [13.7–16.9] g/day for cases respectively (Table 1), considerably higher than reported intakes of 5–6 g/day or lower in the US [50].

Interestingly, rs3877899 (*SEPP1*) genotype has previously been found to be associated with risk of prostate cancer, another hormone-dependent cancer [23]. Although the strongest and most robust risk factors for BC are increased female hormone levels [31], [51], the roles of hormones in BC aetiology are complex; on one hand the production of ROS through redox cycling of the catechol estrogens has been suggested to contribute to breast carcinogenesis by increasing DNA damage, and on the other hand, estrogens have been shown to upregulate antioxidant enzymes (MnSOD and glutathione peroxidases) via the NFκB pathway in animal models [52], [53], [54]. Notably blood Se levels vary through the menstrual cycle with the highest levels coinciding with the peak of estrogen levels [55]. Since SePP1 accounts for the majority of blood Se this suggests that SePP1 levels, and thus Se supply, may respond to estrogen.

eGPx activity has been reported previously to be greater in controls than in breast cancer cases [10]. In the additional work presented here on the same data a more complex relationship between genotype for rs1050450 (*GPX1*), eGPx activity, HRT use and BC risk was revealed. eGPx activity was higher in women currently using HRT compared with former/never users of HRT, suggesting that HRT use raised eGPX activity. This hypothesis is supported by the effect of β-estradiol in increasing both GPx1 protein levels (Figure 1D) and GPx activity in MCF7 cells [43]. The present results indicate that both Se supply and β-estradiol regulate breast cell eGPx activity. In addition, a lower eGPx activity in BC cases compared with controls was only found in homozygous Leu carriers who were current HRT users, suggesting that HRT failed to stimulate an increase in eGPx activity in women who later developed cancer. This could indicate that either 1) low GPx activity predisposes these women to oxidative damage and cancer development, 2) these women require more Se to raise their GPx activity to respond to HRT or 3) Se is diverted towards other tissues (e.g. breast) as an early sign of oxidative stress. The latter explanation is compatible with the observation that estradiol affects Sepp1 and GPx1 mRNA expression and Se tissue distribution in ovarectomized female rats [56].

eGPx activity corresponds mainly to GPx1 activity. Baseline measurements of eGPx activity revealed that rs713041(*GPX4*) also associates with eGPx activity and is compatible with findings that rs713041 affects both the response of lymphocyte GPx1 levels to Se supplementation and the relative binding of proteins to the *GPX1* and *GPX4* 3′UTR [19]. Since protein binding to the SECIS element within the 3′UTR is critical to controlling the relative synthesis of different selenoproteins [18], the present results suggest that women with CC genotype for rs713041 channel Se towards GPx4 synthesis and lowers Se availability for GPx1 synthesis; in contrast, for CT and TT carriers GPx4 competes less for Se and more Se is therefore available for GPx1 synthesis.

Overall, the data suggest a model of GPx1 regulation in which breast *GPX1* expression depends on the combination of dietary Se intake, delivery/distribution of Se (influenced by SNPs in *SEPP1*), factors altering the selenoprotein hierarchy (e.g. SNP in *GPX4* gene), and hormonal regulation of GPx1 expression. The effect on GPx1 expression is further modulated by the presence of the Pro198Leu SNP (rs1050450) altering enzyme activity. We hypothesise that the capacity of an individual to deliver Se to the breast tissue and generate high or low GPx1 activity ultimately determines the susceptibility of their breast tissue to oxidative damage and carcinogenesis. The data indicate that individuals who carry the Leu allele and who have low eGPx activity when using HRT are at increased risk of subsequently developing BC (Figure 2).

Our results suggest that there is a complex interplay between estrogen and Se-dependent antioxidant activity which may play a role in breast carcinogenesis. Genotype for both rs1050450 (*GPX1*) and rs3877899 (*SEPP1*) were found to modulate BC risk and grade in a Danish cohort in which Se intake was previously estimated to be low [42]. Previously genotype for rs713041 (GPX4) has been reported to be associated with prognosis after diagnosis of breast cancer [26]. The present data show that rs713041 also modulates eGPx activity, with TT carriers who later develop BC exhibiting a significantly lower eGPx compared with the ones who did not develop the disease. Thus, we propose that genotypes for both rs713041 (*GPX4*) and rs1050450 (*GPX1*) influence breast GPx1 activity and that this impinges on BC risk and grade. In conclusion, the our data provide evidence that SNPs in *SEPP1* and *GPX1* modulate risk of BC and that eGPx activity is modified by SNPs in *SEPP1, GPX4* and *GPX1* and by estrogens, thus suggesting a role of selenoproteins in BC development. Although earlier work has suggested that antioxidant enzyme SNP genotypes are not useful in screening for human disease [57], the present findings lead us to speculate that the *combination* of selenoprotein genotype and eGPx activity may be a useful biomarker of BC risk in populations where Se intake is relatively low, for example as has been found in European populations [47].
